# Genome Sequences of 22 T1-like Bacteriophages That Infect *Enterobacteriales*

**DOI:** 10.1128/mra.01221-21

**Published:** 2022-04-07

**Authors:** Alyssa Cranston, Parker Danielson, Daniel K. Arens, Austin Barker, Elisabeth K. Birch, Hannah Brown, Emilee Carr, Paige Cero, Jacob Chow, Elisa Correa, Jeremy Dean, Matthew Dunn, Nathaniel Eberhard, Ashley Egbert, Kent Foster, Rochelle Gaertner, Austen Gleave, Alex Gomez, J. Ben Gordon, Evan B. Harris, Corden Heaps, Matthew Hyer, Austin Johnson, Liam Johnson, Minji Kim, Jared L. Kruger, Thomas Leonard, Austin LeSueur, Sophia Lima, Naomi Marshall, Rebecca Moulton, Colleen R. Newey, Daniel Owen, Audra Packard, Alexis Rolfson, Aurora R. Suorsa, Walter Rodriguez, Claudia Sandoval, Ruchira Sharma, Ashley Smith, Carson Sork, Coby Soule, Silvia Soule, Jared Stewart, Tyson Stoker, Daniel W. Thompson, Trever Thurgood, Jamison Walker, Ethan Zaugg, Sherwood R. Casjens, Julianne H. Grose

**Affiliations:** a Department of Microbiology and Molecular Biology, Brigham Young University, Provo, Utah, USA; b School of Biological Sciences, University of Utah, Salt Lake City, Utah, USA; c Division of Microbiology and Immunology, Department of Pathology, University of Utah School of Medicine, University of Utah, Salt Lake City, Utah, USA; Loyola University Chicago

## Abstract

Here, the full genome sequences of 22 T1-like bacteriophages isolated from wastewater are reported. Eight (BlueShadow, Brooksby, Devorator, ElisaCorrea, Reinasaurus, SorkZaugg, Supreme284, ZeroToHero) were isolated on *Citrobacter*, six on Klebsiella (Chell, FairDinkum, HazelMika, Opt-817, P528, PeteCarol), and eight on Escherichia (Fulano1, Mishu, Opt-719, PhleaSolo, Punny, Poky, Phunderstruck, Sadiya).

## ANNOUNCEMENT

Bacteriophage T1 is one of the seven classic T phages, yet the T1-like phages are the least-studied members of this group. This deficit has been attributed to T1’s stability upon drying, which makes it difficult to maintain in the laboratory. Here, we report the isolation and full-genome sequences of 22 T1-like bacteriophages isolated in the undergraduate Phage Hunters Program at Brigham Young University. All 22 bacteriophages infect members of the *Enterobacteriales*.

A total of 22 T1-like bacteriophages were isolated from raw sewage obtained from wastewater treatment plants in the western United States. Bacteriophages were enriched prior to purification by incubation of raw sewage with the bacterial host in LB medium at 37°C. The resulting lysates were centrifuged to remove cells and debris, and the supernatant was incubated for 30 min with fresh bacteria and plated in LB top agar. Single plaques were isolated by incubation with fresh bacterial overnight culture and subsequent plating in LB top agar. Single plaque purification was repeated a minimum of three times prior to growing a liquid culture from a single plaque. Following removal of bacteria and debris by centrifugation, DNA was isolated from the resulting lysate (>10^8^ PFU/mL) using the Norgen Biotek phage DNA isolation kit (Canada). Phage genomic DNA was prepped for 150-bp paired-end Illumina iSeq sequencing using the NEBNext Ultra II DNA kit or for 250-bp paired-end Illumina HiSeq 2500 or 150-bp paired end MiSeq sequencing using the Illumina TruSeq DNANano kit (Table 1). All contigs were assembled *de novo* using Geneious v. R11 for HiSeq data or v. 8.0.5 for iSeq or MiSeq data (1) and subsequently annotated using DNA Master (2) and GeneMarkS (3). All software was used at default settings. All 22 genomes circularized upon assembly and were assigned to the T1-like bacteriophage cluster previously described (4) using dotplot (5) and BLASTn analysis (6).

**TABLE 1:** Sequencing summary and basic properties of 22 T1-like *Enterobacteriales* phages

Phage name	GenBank accession no.	SRA accession no.	Total no. of reads	Fold coverage range (mean)	Length (bp)	GC content (%)	Tax^*a*^^,^^*b*^	Sewage sample GPS (N, W)
vB_KaeD_HazelMika	OL539457	SRR17231386	238,191	770–2478 (1122.7)	51,552	49.6	*Vi*	38.2492, 122.0405
vB_EcoD_Fulano1	OL539459	SRR17231380	135,106	204–645 (394.3)	51,759	43.9	*Ha*	40.1374, 111.6518
vB_EcoD_Phunderstruck	OL539446	SRR17231359	77,290	113–389 (227.7)	51,274	44.6	*Wa*	40.6150, 111.9290
vB_EcoD_Poky	OL539445	SRR17231362	39,391	47–179 (115.8)	51,374	44.9	*Wa*	33.1167, 117.3208
vB_EcoD_Opt-719	OL539451	SRR17231351	415,000	110–35 (172)	48,302	44.7	*Ro*	39.7392, 104.9903
vB_EcoD_Sadiya	OL539467	SRR17231355	3,357	4–47 (17.2)	48,403	44.7	*Ro*	33.1959, 117.3795
vB_EcoD_PhleaSolo	OL539447	SRR17231350	67,520	1,503–4,637 (2,162.5)	48,301	44.7	*Ro*	39.7392, 104.9903
vB_KpnD_P528	MW021764	SRR10580542	2,754	1–19 (7.5)	51,895	51.3	*We*	38.2578, 122.0543
vB_KpnD_Opt-817	OL539450	SRR17231352	568,989	1,855–4,720 (2871.7)	48,988	51.5	*We*	40.1150, 111.6549
vB_KpnD_Chell	OL539472	SRR17231357	274,748	1–2,630 (1,229.2)	51,779	51.6	*We*	40.1652, 111.6108
vB_KpnD_PeteCarol	OL539448	SRR17231371	31,600	38–174 (97.3)	49,054	50.9	*We*	41.0919, 112.0686
vB_KpnD_FairDinkum	OL539460	SRR17231376	83,417	204–790 (405.7)	49,111	50.7	*We*	40.7418, 73.9893
vB_CfrD_Supreme284	MW021750	SRR17231367	41,557	42–211 (126.4)	49,439	42.9	*T1*	40.8167, 111.9321
vB_CfrD_ElisaCorrea	OK499973	SRR17231372	22,111	26–121 (67.1)	49,522	42.9	*T1*	40.2779, 111.9302
vB_CfrD_Reinasaurus	OL539465	SRR17231369	80,771	157–436 (246.1)	48,103	42.9	*T1*	40.2969, 111.6946
vB_CfrD_ZeroToHero	MW021748	SRR17231366	30,748	49–151 (92.8)	49,802	42.8	*T1*	40.7188, 111.8883
vB_CfrD_Brooksby	OL539443	SRR17231384	80,874	158–398 (244)	50,071	42.8	*T1*	40.2134, 111.6500
vB_CfrD_BlueShadow	OL539462	SRR17231377	18,855	19–122 (57.5)	49,619	42.8	*T1*	40.2779, 111.7382
vB_CfrD_Devorator	OL539461	SRR17231383	64,357	42–369 (196)	49,712	42.9	*T1*	33.1108, 117.3195
vB_CfrD_SorkZaugg	OL539466	SRR17231374	70,999	95–328 (214.4)	50,022	42.8	*T1*	40.6155, 111.9245
vB_EcoD_Punny	OL539444	SRR17231382	107,437	59–246 (132.4)	49,765	42.8	*T1*	40.2143, 111.6533
vB_EcoD_Mishu	OK499984	SRR17231381	43,568	174–641 (364.8)	49,715	42.9	*T1*	40.8893, 111.8807

a Taxonomy from BLASTN taxonomy output on the NCBI database. *Vi*, *Vilniusvirus*; *Ha*, *Hanrivervirus*; *Ro, Rogunavirus*; *Wa, Warwickvirus*; *We*, *Webervirus*; *T1*, *T1svirus*.

b Shading is to highlight the taxonomy groups.

The T1-like phage cluster corresponds to the *Drexlerviridae* family designated by the International Committee of Taxonomy of Viruses (ICTV). Taxonomy of the newly isolated phages was determined with the taxonomy output of BLASTn at the NCBI website, where all 22 phages were assigned to genera in the *Drexlerviridae*. Of the 22 bacteriophages reported here, five were isolated using Klebsiella pneumoniae ATCC 10031 and were assigned to the genus *Webervirus* (Chell, FairDinkum, P528, PeteCarol, Opt-817). HazelMika was isolated on Klebsiella aerogenes ATCC 13047 and was assigned to *Vilniusvirus*. Eight bacteriophages were isolated on Escherichia coli BW25113 (7) and fall into four groups, *Hanrivervirus* (Fulano1), *Rogunavirus* (Opt-719, PhleaSolo, Sadiya), *Warwickvirus* (Poky and Phunderstruck), and *Tlsvirus* (Punny and Mishu). Eight phages isolated on Citrobacter freundii (Braak) Werkman and Gillen (ATCC 8090) reside in the genus *Tlsvirus* (Brooksby, BlueShadow, Devorator, ElisaCorrea, Reinasaurus, SorkZaugg, Supreme284, ZeroToHero). Further characterization of these T1-like phages will aid in the understanding of this abundant and diverse phage cluster, whose members are capable of infecting a wide range of bacterial hosts.

### Data availability.

The accession numbers for all 22 bacteriophages are listed in Table 1.
